# Assessment of the risk of exposure to cadmium and lead as a result of the consumption of coffee infusions

**DOI:** 10.1007/s12011-020-02332-3

**Published:** 2020-08-13

**Authors:** Anna Winiarska-Mieczan, Katarzyna Kwiatkowska, Małgorzata Kwiecień, Ewa Zaricka

**Affiliations:** 1 Department of Bromatology and Food Physiology, University of Life Sciences in Lublin, Akademicka 13, 20-950 Lublin, Poland; 2 State Scientific-Research Control Institute of Veterinary Medical Products and Feed Additives, Lviv, Ukraine

**Keywords:** Coffee infusions, Cadmium, Lead, Risk assessment

## Abstract

The paper aimed to analyse the safety of drinking coffee by adult Poles in terms of Pb and Cd content. The degree to which Cd and Pb passed from coffee grounds into the coffee infusion was also examined. Twenty-three samples of natural coffee were examined. The content of metals was determined using the ICP method. On average, dry coffee contained ca. 0.004 μg Cd and 0.05 μg Pb per 1 g, and 95.5% Cd and 94% Pb passed into the infusion. Drinking coffee supplies these metals in the amount of less than 2% TWI (tolerable weekly intake) for Cd and BMDL (benchmark dose lower confidence limit) for Pb. In the presented studies, the values of CDI (chronic daily intake), THQ (target hazard quotient) and HI (hazard index) indicators were lower than 1, which means that the risk of developing diseases connected with chronic exposure to Cd and Pb consumed with coffee must be evaluated as very low. The content of Cd and Pb in the analysed coffee infusions was very low, so drinking coffee does not pose a risk for consumers in terms of the content of these metals. However, it must be remembered that no threshold limits for toxic metal consumption exist because these metals accumulate in the body for a long time. The studies presented here also showed a low (*r* = 0.26) but still a positive correlation between the content of Pb in coffee and the degree (%) to which Pb passed into the infusion. This problem should be thoroughly investigated.

## Introduction

Coffee, next to tea, is one of the most popular drinks in the world [1]. It is a source of antioxidants including caffeine, phenolic compounds and diterpenes. Results of studies suggest that drinking coffee can increase the level of glutathione and improve the protection of the body against DNA damage, in particular, if consumed regularly [1]. It was demonstrated that drinking coffee decreased the risk of developing breast cancer, prostate and colorectal cancer, which is attributed to the presence of antioxidants [2, 3]. It is also suggested that drinking coffee decreases the risk of developing chronic diseases such as type 2 diabetes and Parkinson’s [4, 5]. According to surveys, 95.2% of adult Poles drink coffee compared with 61% of Italians and about 40% of Spaniards [6, 7]. Statistically, in 2017 in Poland, the consumption of coffee amounted to 2.16 kg per person [8]. In Poland, the most popular type of coffee is non-instant coffee (ground and grained). This is a choice of more than 50% of consumers, preceding instant coffee and coffee mixes [6]. Most often, Poles drink 1–3 cups of coffee a day [9, 10]. However, one should not drink more than 5 cups (1 cup = 150 ml = 80 mg caffeine) a day due to its possible negative effect on the cardiovascular system (increased LDL-chol and total cholesterol levels due to diterpenoid alcohols), problems falling asleep (caffeine), pregnancy (caffeine intake of > 300 mg per day proved negative effect on the duration of pregnancy and weight at birth) and increased secretion of gastric acid and bile, which exacerbates peptic ulcer disease and hyperacidity [1, 11–13].

Apart from antioxidants and other bioactive compounds, coffee contains carbohydrates, lipids, nitrogen compounds, vitamins and minerals, including toxic elements such as cadmium (Cd) and lead (Pb) [12, 14, 15]. The presence of toxic metals in food is a global problem. Their primary source for humans is the food of plant origin [16, 17]. Although, according to available literature and own studies, the content of Cd and Pb in food products normally does not exceed acceptable standard levels, due to the fact that these metals are capable of accumulating in tissues and have a long half-life: 5–30 years for Cd and from 30 days (in soft tissue) to 10 years (in bones) for Pb [18], their regular supply, even in small amounts, is dangerous. These metals display mutagenic, teratogenic, carcinogenic and embryotoxic effects [19]. In 2012, EFSA reduced the tolerable intake level for Cd and Pb. The TWI (tolerable weekly intake) for Cd was determined at the level of 2.5 μg kg^−1^ of body weight per week [16], whereas the BMDL (benchmark dose lower confidence limit) for Pb was BMDL_01_—10.5 μg kg^−1^ of body weight per week—and BMDL_10_—4.4 μg kg^−1^ of body weight per week [17]. The paper aimed at analysing the safety of drinking coffee by adult Poles in terms of Pb and Cd content. The degree to which Cd and Pb passed from coffee grounds into the coffee infusion was also examined. The presented results are a part of the project aiming to estimate the intake of minerals (toxic and essential) in the Polish population.

## Material and methods

### Study material

Twenty-three samples of natural coffee were examined (Table 1). The products were purchased in August 2017 from local groceries, still within their shelf life. Before the analyses, the coffee was stored in original, tightly sealed packages at room temperature.

**Table 1:** Characteristic of the analysed products

	Coffee form	Coffee varieties	Trademark	Size of package, g	Annotation	Origin	Made in
1	Beans	Arabica + Robusta	A	1000		South America, Asia, Africa	Poland
2	Beans	No data	B-1	1000		No data	Poland
3	Ground	No data	B-2	250		No data	Poland
4	Ground	Robusta	C	400		India	Poland
5	Beans	Arabica	D-1	500		Brazil	Holland
6	Beans	Arabica + Robusta	D-2	1000		No data	Holland
7	Ground	Arabica	D-1	500		Columbia	Holland
8	Ground	Arabica	D-3	500		Brazil	Holland
9	Ground	Arabica	G-1	250	Bio, Fair Trade	Papua New Guinea, Peru, Mexico	Italy
10	Ground	Arabica	G-2	250		Brazil	Italy
11	Beans	Arabica + Robusta	G-3	1000		South America, Indonesia	Italy
12	Ground	Arabica + Robusta	I	250		South America, Indonesia	Germany
13	Ground	Robusta	J-1	500		Vietnam	Germany
14	Ground	Robusta	J-2	250		India	Germany
15	Ground	Robusta	J-2	100		India	Germany
16	Ground	Arabica	J-2	500		Brazil	Germany
17	Beans	Arabica	J-3	1000	Fair Trade	Bolivia, Peru, Ecuador, Nicaragua	Germany
18	Ground	Arabica	K-1	500		Brazil	Germany
19	Ground	Arabica	K-2	500		South America	Germany
20	Beans	Arabica	K-3	500		South America	Germany
21	Beans	Arabica + Robusta	K-4	500		South America, Indonesia	Germany
22	Ground	Arabica	L	500		Brazil	Germany
23	Ground	Arabica	M	227	Fair Trade	Peru, Nicaragua	England

### Preparation of samples for analyses

Grained coffee was ground in a laboratory grinder with plastic blades. Ground coffee was mixed by hand. Coffee infusions were prepared as follows: 6 g of ground coffee was poured with 100 ml of drinking water with a temperature of 95–100 °C; after 10 min, the solutions were drained through the Whatman drain. The resulting coffee grounds were dried in a drier at a temperature of 65 °C for 24 h. Afterwards, they were pulverised in a laboratory grinder with plastic blades. The analyses covered both fresh ground coffee and coffee grounds remaining after coffee brewing.

### Chemical analyses

The analysed material was manually mixed. Samples weighing ca. 3 g were weighed in 3 replications into previously heat sterilised china crucibles and then subjected to dry mineralisation in a muffle furnace at a temperature of 450 °C. The oxidant was hydrogen peroxide. The mineralisate was dissolved in 10 ml of 1 M HNO_3_ [20, 21]. The content of cadmium and lead was determined using ICP (inductively coupled plasma mass spectrometry) in a Varian 820 MS spectrometer (Varian, Melbourne, Australia). The parameters for determination and control of correct analyses were included in Table 2. The calibration curve was drawn using the models:

- Cd: standard characterised by 99.999% purity used to prepare solutions with the concentration of 0.2; 0.4; 1; 2; 4; 10 μg of Cd L^−1^; the solutions were prepared in 1% ultra-pure nitric acid (V).
- Pb: standard characterised by 99.999% purity used to prepare solutions with the concentration of 0.1; 0.2; 0.5; 1; 2; 5 μg of Pb L^−1^; the solutions were prepared in 1% ultra-pure nitric acid (V).

**Table 2:** Measurement parameters and validation data for the determination of Cd and Pb levels by ICP-MS

	Cd	Pb
Mass monitored	114	206; 207; 208
Plasma gas	Argon	Argon
Plasma gas flow, L min^−1^	18	18
Nebuliser gas flow, L min^−1^	1	1
Auxiliary gas flow, L min^−1^	1.70	1.70
Sampling depth, mm	5	5
RF power, kW	1.37	1.37
Limit of detection LOD, μg kg^−1^	0.004	0.005
Limit of quantification LOQ, μg kg^−1^	0.010	0.030
Quality control		
Blank sample	1 M HNO_3_	1 M HNO_3_
Certified reference material (1)	INCT-TL-1 (tea leaves)	INCT-TL-1 (tea leaves)
Certified reference material (2)	INCT-MPH-2 (mixed Polish herbs)	INCT-MPH-2 (mixed Polish herbs)
Certified element concentration in CRM 1		
Certified, mg kg^−1^	0.030	1.78
Observed, mg kg^−1^	0.029	1.76
Recovery rate, %	98	99
Certified element concentration in CRM 2		
Certified, mg kg^−1^	0.199	2.16
Observed, mg kg^−1^	0.189	2.22
Recovery rate, %	95	103
Precision, %	6.04	6.07
Replicates	3	3

Each chemical analyses was repeated 3 times. The accuracy of determination was verified using a blind test (1 M HNO_3_) and two certified reference materials (CRM): INCT-TL-1 Tea leaves (containing 0.030 mg Cd and 1.78 mg Pb per 1 kg) and INCT-MPH-2 Mixed Polish herbs (containing 0.199 mg Cd and 2.16 mg Pb per 1 kg).

### Reagents and reference materials

Hydrogen peroxide H_2_O_2_ (30% pure) and nitric acid HNO_3_ (65% ultra-pure) were purchased from POCH S.A. (Poland). Deionised water used for dilution was made in our laboratory (Hydrolab Poland, Gdańsk). The Cd and Pb standards were purchased from Merck (Germany). Certified reference materials INCT-TL-1 and INCT-MPH-2 were obtained from the Institute of Nuclear Chemistry and Technology (Warsaw, Poland).

### Calculations

Based on the difference in the content of Cd and Pb in coffee grounds, the degree (%) to which those metals passed into the infusion was calculated prior to after coffee brewing.

The safety of drinking coffee for adult Poles was estimated on the grounds of (1) calculation of the percentage of Cd and Pb intake in comparison with the acceptable level proposed by EFSA [16, 17], (2) calculation of parameters describing the risk of development of cancer and (3) calculation of parameters describing the risk of development of non-carcinogenic diseases. Three consumption patterns were taken into account in the calculations: 1 cup, 2 cups or 3 cups a day for 365 days in a year because such amounts of coffee in Poland are drunk by ca. 80% of coffee drinkers [9, 10].

1. Percent of tolerable dose:

Estimated weekly intake (EWI) of Cd and Pb was calculated according to the formula [22]:

$$ EWI=\frac{MWC\times metal\ level}{100} $$

where MWC is the mean weekly consumption of coffee (one, two or three cups).

Tolerable weekly intake % (TWI) was calculated according to the formula [22]:

$$ \% TWI=\frac{EWI_{Cd}\times 100}{TWI} $$

The value adopted for TWI was 2.5 μg Cd kg^−1^ of body weight per week [16].

Benchmark dose lower confidence limit % (BMDL) was calculated according to the formula [22]:

$$ \% BMDL=\frac{EWI_{Pb}\times 100}{BMDL} $$

The value adopted for BMDL: two values suggested by the European Food Safety Authority (EFSA) were calculated per 1 week: BMDL_01_—10.5 μg Pb kg^−1^ of body weight per week—and BMDL_10_—4.4 μg Pb kg^−1^ of body weight per week [17].

The mean body weight was assumed as 70 kg.

(2) Cancer risks parameters

Chronic daily intake (CDI) of Cd or Pb was calculated according to the formula [23, 24]:

$$ \mathrm{CDI}=\frac{\mathrm{ED}\mathrm{I}\times \mathrm{EFr}\times {\mathrm{ED}}_{\mathrm{tot}}\ }{\mathrm{body}\ \mathrm{weight}\times \mathrm{AT}} $$

where EDI is the estimated daily intake of Cd and Pb, calculated on the basis of the mean weekly consumption of coffee (one, two or three cups) and mean level of Cd and Pb; EFr is the days of exposure frequency (365 per year); ED_tot_ is the exposure duration (years)—since in Poland regular coffee drinkers are adults only, it was assumed that the time of exposure was calculated from 18 to 74 years of age (74 years—average life span in Poland), which is 56 years; AT is the period of exposure (365 per year).

CSF is a cancer slope factor which is the risk produced by a lifetime average dose of 1 mg kg^−1^ BW per day and is contaminant specific.

(3) Non-carcinogenic risks parameters

Target hazard quotient (THQ) was calculated according to the formula [23]:

$$ \mathrm{THQ}=\mathrm{CDI}/\mathrm{RfD} $$

where CDI is the chronic daily intake of Cd or Pb.

RfD (reference dose) for Cd is 1 μg kg^−1^ of body weight per day, whereas, for Pb, it is 3.5 μg kg^−1^ of body weight per day [25].

When THQ is higher than 1, it is assumed that there is a significant risk of developing negative effects on health resulting from chronic exposure to Cd and/or Pb [26].

Hazard index (HI) was calculated according to the formula [23]:

$$ \mathrm{HI}={\mathrm{THQ}}_{\mathrm{Cd}}+{\mathrm{THQ}}_{\mathrm{Pb}} $$

### Statistical analysis

The mean content of Cd and Pb was calculated for each sample (three weighing replications × 3 replications of chemical analysis). A statistical analysis of the results (average value, minimum and maximum value, standard deviation, median, 75 and 25 percentile) was carried out using Statistica 13.1 software. Statistically significant differences (*P* < 0.05) were computed by single factor analysis of variance (ANOVA), using the Duncan test. The correlation between the content of Cd and Pb in coffee and the degree (%) to which they passed into the infusion was calculated using Pearson’s method (Statistica 6.0 software).

## Results

### Content of Cd and Pb in coffee

**Fig. 1:**
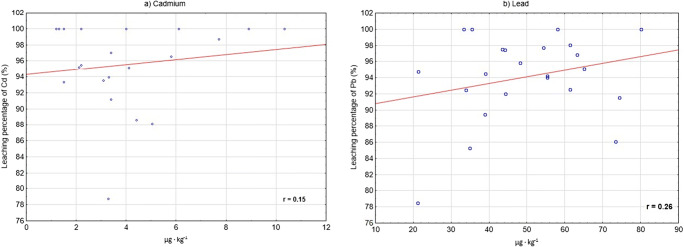
Correlation between the content of Cd (**a**) and Pb (**b**) in coffee and the degree (%) to which they pass into the infusion

Dry coffee prior to brewing contained from 1.204 to 10.33 μg Cd per 1 kg (Tables 3 and 4). The mean content of Cd in the analysed samples was 3.784 μg (± 2.464) per 1 kg. Coffee grounds contained from < LOQ to 0.698 μg Cd per 1 kg; in 35% of samples, the level of Cd was lower than determinable with the applied method (LOQ = 0.01 μg kg^−1^). About 79 to 100% (on average 95.5%) of Cd present in the output material passed into the infusion; the infusion contained, on average 3.613 μg kg^−1^ (range 1.2–10.33 μg). A very low positive correlation *r* = 0.15 was identified between the content of Cd in coffee and the degree (%) to which Cd passed into the infusion (Fig. 1a). On average, dry coffee prior to brewing contained ca. 49.6 μg kg^−1^ Pb (range 21.22–80.06 μg kg^−1^), whereas coffee grounds < LOQ—10.2 μg kg^−1^. In 17% of coffee ground samples, the level of Pb was lower than determinable using the analytical method applied (LOQ = 0.03 μg kg^−1^). From nearly 79 to 100% (on average 94%) of Pb passed into the infusion, the infusion contained, on average, 46.86 μg kg^−1^ (range 16.66–80.06 μg). A low positive correlation *r* = 0.26 was identified between the content of Pb in coffee and the degree (%) to which Pb passed into the infusion (Fig. 1b).

**Table 3:** Content of Cd and Pb in dry ground coffee (before brewing), dregs and infusions (*n* = 23), μg kg^−1^

	Dry coffee		Dregs		Infusions		Leaching percentages of Cd and Pb	
	Cd	Pb	Cd	Pb	Cd	Pb	Cd	Pb
1	5.041^E^	73.37^F^	0.600^F^	10.20^I^	4.443^D^	63.17^F^	88^B^	87^A^
2	10.33^I^	74.37^F^	< LOQ^A^	6.280^H^	10.33^H^	68.09^F^	100^C^	92^A, B^
3	1.204^A^	34.84^B^	< LOQ^A^	5.134^G^	1.203^A^	29.71^C^	100^C^	85^A^
4	3.991^D^	38.04^B, C^	< LOQ^A^	2.143^C^	3.990^D^	36.89^C, D^	100^C^	95^B^
5	8.883^H^	43.47^C^	< LOQ^A^	1.087^B^	8.882^G^	42.38^D^	100^C^	97^B^
6	3.289^C^	80.06^G^	0.702^G^	< LOQ^A^	2.593^B, C^	80.06^G^	78^A^	100^B^
7	1.502^A^	44.21^C^	< LOQ^A^	1.120^B^	1.502^A, B^	43.09^D^	100^C^	97^B^
8	2.100^B^	33.87^B, C^	0.100^B^	2.549^C^	2.000^B^	31.32^C^	95^B, C^	92^A, B^
9	3.411^C, D^	58.12^D, E^	0.313^D^	< LOQ^A^	3.100^C^	58.12^E, F^	91^B, C^	100^B^
10	2.204^B^	61.35^E^	0.122^B^	1.223^B^	2.084^B^	60.13^F^	95^B, C^	98^B^
11	1.273^A^	55.41^D^	< LOQ^A^	3.320^E^	1.272^A^	52.09^E^	100^C^	94^A, B^
12	2.100^B^	61.35^E^	< LOQ^A^	4.581^F^	1.994^B^	56.77^E, F^	95^B, C^	93^A, B^
13	3.122^C^	54.36^D^	0.189^C^	1.250^B^	2.932^C^	53.11^E^	94^B, C^	98^B^
14	7.703^G^	33.35^B^	0.133^B^	< LOQ^A^	7.573^F^	33.35^C^	98^B, C^	100^B^
15	6.111^F^	44.36^C^	< LOQ^A^	3.561^D^	6.110^E^	40.80^D^	100^C^	92^A, B^
16	1.489^A^	55.36^D^	0.100^B^	3.210^D^	1.389^A^	52.15^E^	93^B, C^	94^A, B^
17	2.110^B^	21.36^A^	0.122^B^	1.122^B^	1.980^B^	20.24^B, C^	95^B, C^	95^A, B^
18	3.311^C^	65.12^E^	0.191^C^	3.220^D^	3.122^C^	6.900^A^	94^B, C^	95^A, B^
19	4.089^D^	21.22^A^	0.210^C^	4.558^E^	3.881^C^	16.66^B^	95^B, C^	78^A^
20	2.210^B^	35.48^B^	< LOQ^A^	< LOQ^A^	2.200^B^	35.48^C, D^	100^C^	100^B^
21	3.401^C, D^	48.22^C^	0.103^B^	2.010^C^	3.321^C^	46.21^D, E^	97^B, C^	96^B^
22	5.824^F^	63.22^E^	0.222^C^	2.011^C^	5.602^E^	61.21^F^	97^B, C^	97^B^
23	4.350^D^	38.95^B, C^	0.484^E^	4.123^F^	3.874^C^	34.83^C^	89^B^	89^A, B^

Average values for 3 replications

^A, B^Means with different superscripts in the same column differs significantly at *P* < 0.05 by Duncan’s test; LOQ Cd = 0.010 μg kg^−1^; LOQ Pb = 0.030 μg kg^−1^

**Table 4:** Results of coffee analysis (*n* = 23)

	Dry coffee	Dregs	Infusions	Leaching percentages of Cd and Pb
Cd, μg kg^-1^				
Mean	3.784	0.156	3.613	95.49
Maximum	10.33	0.698	10.33	100.0
Minimum	1.204	< LOQ	1.200	78.79
Median	3.300	0.100	3.101	95.45
SD	2.464	0.197	2.471	5.162
Variance analysis	0.607	0.001	0.609	26.65
Percentile				
75%	4.720	0.200	4.222	100.0
25%	2.100	< LOQ	2.000	93.74
Percent of samples < LOQ	0%	35%		
Pb, μg kg^-1^				
Mean	49.59	2.726	46.86	94.07
Maximum	80.06	10.20	80.06	100.0
Minimum	21.22	< LOQ	16.66	78.51
Median	48.22	2.143	46.21	94.76
SD	16.27	2.412	15.85	5.332
Variance analysis	26.47	5.819	25.16	28.43
Percentile				
75%	61.35	3.840	59.12	97.60
25%	37.22	1.120	35.16	92.22
Percent of samples < LOQ	0%	17%		

Average values for samples, each in 3 replications; *SD*, standard deviation; *LOQ*, limit of quantitation; LOQ Cd = 0.01 μg kg^−1^; LOQ Pb = 0.03 μg kg^−1^

### Coffee drinking safety

Data concerning the estimated safety of drinking coffee infusions, taking into account three consumption patterns (1, 2 or 3 cups of coffee a day), is presented in Table 5.

**Table 5:** Safety of coffee for consumption

	Cd	Pb
Pattern 1: drinking 1 cup of coffee a day		
EWI, μg^1^	0.156	1.968
% TWI^A, B^	0.089	
% BMDL_01_^A, C^		0.268
% BMDL_10_^A, D^		0.639
CD^2^	0.022	0.281
THQ^3^	0.022	0.080
HI^4^	0.103	
Pattern 2: drinking 2 cups of coffee a day		
EWI, μg^1^	0.312	3.936
% TWI^A, B^	0.178	
% BMDL_01_^A, C^		0.541
% BMDL_10_^A, D^		1.278
CDI^2^	0.045	0.562
THQ^3^	0.045	0.161
HI^4^	0.205	
Pattern 3: drinking 3 cups of coffee a day		
EWI, μg^1^	0.468	5.715
% TWI^A, B^	0.267	
% BMDL01^A, C^		0.778
% BMDL10^A, D^		1.856
CDI^2^	0.067	0.816
THQ^3^	0.067	0.233
HI^4^	0.300	

^1^*EWI*, estimated weekly intake calculated on the basis of the mean weekly consumption of coffee infusions and mean level of Cd and Pb

^2^Chronic daily intake calculated on the basis of the mean weekly consumption of coffee, mean level of Cd and Pb and exposure duration

^3^Target hazard quotient calculated on the basis of the chronic daily intake of Cd or Pb

^4^Hazard index is the sum of THQ for Cd and Pb

^A^Mean body weight was assumed as 70 kg

^B^TWI, 2.5 μg Cd per kg of body weight per week [16]

^C^BMDL01, 10.5 μg Pb per kg of body weight per week [17]

^D^BMDL10, 4.4 μg Pb per kg of body weight per week [17]

#### Pattern 1: 1 cup of coffee a day

The estimated weekly intake (EWI) of Cd with coffee infusion is 0.156 μg, which accounts for about 0.09% TWI. The value of CDI_Cd_ and THQ_Cd_ indicators is identical and it amounts to 0.022. The estimated weekly intake of Pb is 1.968 μg, which corresponds to ca. 0.27% BMDL_01_ and ca. 0.64% BMDL_10_. The value of CDI_Pb_ = 0.281, whereas that of THQ_Pb_ = 0.08. The HI risk factor (Cd + Pb) is 0.103.

#### Pattern 2: 2 cups of coffee a day

EWI of Cd with coffee is 0.312 μg, which accounts for 0.18% of TWI. The value of CDI_Cd_ and THQ_Cd_ indicators is 0.045 each. EWI of Pb is ca. 3.94, which corresponds to 0.54% BMDL_01_ and nearly 1.3% BMDL_10_. The value of CDI_Pb_ = 0.56, whereas THQ_Pb_ = 0.16. The HI risk factor equals 0.205.

#### Pattern 3: 3 cups of coffee a day

EWI of Cd with infusion is less than 0.5 μg, which accounts for about 0.27% TWI. The values of CDI_Cd_ and THQ_Cd_ indicators are 0.067 each. EWI of Pb was equal to 5.715 μg, which corresponds to about 0.78% BMDL_01_ and about 1.86% BMDL_10_. The value of CDI_Pb_ = 816, whereas THQ_Pb_ = 233. The HI risk factor equals 0.3.

## Discussion

In the presented studies of this author, dry coffee contained on average nearly 3.8 μg Cd and ca. 50 μg Pb per 1 kg of the natural product, which accounts for ca. 0.004 μg Cd and 0.05 μg Pb per 1 g. As 95.5% Cd and 94% Pb passed into the infusion, the infusion contained on average 0.0037 μg Cd and ca. 0.047 μg Pb per 1 g. Considering the consumption of coffee infusion (1, 2 or 3 cups a day), an adult Pole consumes less than 0.5 μg Cd and nearly 6 μg Pb per day. Nędzarek et al. [14] examined the content of, among other elements, Pb in infusions of coffee purchased in Poland (*n* = 4), Lebanon (*n* = 1), Brazil (*n* = 3) and in Bosnia and Herzegovina (*n* = 3). These studies showed that infusions contained from 0.615 (Polish coffee) to 1.24 μg (Bosnia and Herzegovina) Pb per 1 g. According to those authors, in Poland, a person drinking 2.4 kg of coffee on an annual basis simultaneously consumes 1.48–2.43 mg Pb, which is slightly more than 4 μg Pb per day, whereas, in Bosnia and Herzegovina, it is ca. 33 μg Pb per day (12 mg per year).

Grembecka et al. [27] in 120 samples of different types of coffee, including 75 samples of ground coffee and 27 of instant coffee, found the presence of Cd and Pb in amounts lower than determinable using the applied method of analysis (LOD Cd = 0.003 mg 100 g^−1^, LOD Pb—0.01 mg 100 g^−1^). Gebretsadik et al. [28] in Ethiopian ground coffees found that the level of Cd was lower than 0.01 μg g^−1^, whereas that of Pb < 0.04 μg g^−1^. Similarly, Ashu and Chandravanshi [29] found that both in dry grains and infusions of Ethiopian coffee (*n* = 3), the level of Cd and Pb was lower than LOD. Studies in Brazil demonstrated that the level of Cd in roasted ground coffee (*n* = 15) was < 0.025 μg g^−1^, whereas the level of Pb ranged from 0.14 to 2.59 μg g^−1^ [15]. In as many as 8 samples, the level of Pb exceeded the maximum limit accepted by Brazilian legislation 0.5 μg g^−1^ [15]. According to other Brazilian studies [30], ground coffees contained 0.03–0.1 mg Cd and 0.025–1.58 mg Pb per 1 kg. The same authors found that the coffees were not safe in terms of the content of Pb; in 75% of 50 analysed samples, it contained more Pb than acceptable in Brazil, whereas, in 86% of samples, it exceeded the limit in the European Union (0.2 μg kg^−1^). In addition, da Silva et al. [30] found that the degree of extraction of Cd into the infusion is 26% and that of Pb is less than 47%; the values were considerably lower than measured by the present author. Instant coffees drunk in India contained 0.001–0.03 μg g^−1^ Cd and 0.02–0.2 μg g^−1^ Pb [31]. Studies carried out in Saudi Arabia showed that dry coffee contained 0.053 μg Pb g^−1^ [32]. In turn, Santos et al. [33] found that in coffee grains, the average content of Cd < 0.1 μg g^−1^, whereas that of Pb < 2.6 μg g^−1^. Turkish studies showed that green coffee grains contained on average 0.005 μg g^−1^ Cd (0.003–0.006 μg g^−1^) and 0.12 μg g^−1^ Pb (0.06–0.3 μg g^−1^) [34]. Those authors recount that up to 84% of Cd and up to 82.6% of Pb pass into the infusion, which is dependent on the coffee brewing method only (Turkish method—cooking—leaches more minerals, including toxic elements—except for Pb, than pouring with boiling water as practised in Poland), but it is not dependent on the type of coffee. Studies by Anderson et al. [35] also showed that Pb can be leached from vessels into the ground coffee infusion—the highest amount of Pb passes from ceramic cups.

In the presented studies, the values of CDI, THQ and HI indicators for all the assumed patterns were lower than 1, which means that the risk of developing diseases connected with chronic exposure to Cd and Pb consumed with coffee must be evaluated as very low. Coffee drinking safety is also confirmed by the degree of coverage of the tolerable intake level of Cd and Pb recommended by EFSA [16, 17]. According to the studies of the present author, drinking 3 cups of coffee a day contributes to supplying these metals in the amount of less than 0.3% TWI (Cd) and less than 2% BMDL (Pb). According to Şemen et al. [34], drinking 2 cups of coffee a day contributes to Cd intake amounting to 0.01–0.06% PTWI and Pb intake amounting to 0.03–0.38% PTWI, depending on the type of coffee and content of toxic metals. Suseela et al. [31] found that drinking instant coffee contributes to intake of Cd amounting to 1.1% and that of Pb amounting to 0.7% of the acceptable limit in India. Pigozzi et al. [15] recount that 1 cup (50 ml) of Brazilian ground coffee infusion contains maximum 2.835 μg Pb, which accounts for 0.21 to 4.54% of the acceptable limit (that is 25 μg kg^−1^ of body weight), while the content of Cd in those coffees was lower than LOD.

To sum up, the content of Cd and Pb in the analysed coffee infusions was very low. However, it must be remembered that no threshold limits for toxic metal consumption exist because these metals accumulate in the body for a long time; in the case of Cd and Pb, it is even 30 years [18], whereas their largest amounts accumulate in organs in charge of detoxicating processes (liver and kidneys) and in the brain [19, 36], leading to their damage and dysfunction. Nędzarek et al. [14] mention the level of Pb in coffee; despite it was low in their studies, those authors suggest that the content of Pb in coffee should be monitored regularly because it is higher than the content of Cd and can accumulate in tissues. Studies involving rats showed that during complex exposure (Cd + Pb), Pb accumulates in the organs to a higher degree than Cd (0.6% vs 0.48% in adults and 0.5% vs 0.7% in a younger population) [19, 36]. Lead is absorbed to a higher extent by the gastrointestinal tract than Cd after oral intake (10–50%, 1–8%) [37, 38]. In the presented study of this authors, an alarming signal is CDI_Pb_ close to 1. It must be taken into account that some authors found that the Pb level was higher than acceptable in 75% of the analysed samples [15, 30]. The studies presented here also showed a low (*r* = 0.26) but still, a positive correlation between the content of Pb in coffee and the degree (%) to which Pb passed into the infusion. This problem should be thoroughly investigated.

## Conclusions

The content of Cd and Pb in the analysed coffee infusions was very low, so drinking coffee does not pose a risk for consumers in terms of the content of these metals. However, it must be remembered that no threshold limits for toxic metal consumption exist because these metals accumulate in the body for a long time; in the case of Cd and Pb, it is even 30 years.
